# Circulating tumor DNA for personalized lung cancer monitoring

**DOI:** 10.1186/s12916-017-0921-6

**Published:** 2017-08-17

**Authors:** Clare Fiala, Eleftherios P. Diamandis

**Affiliations:** 1 Department of Pathology and Laboratory Medicine, Mount Sinai Hospital, 60 Murray St. Box 32, Floor 6, Rm L6-201, Toronto, ON MST 3L9 Canada; 2 Department of Laboratory Medicine and Pathobiology, University of Toronto, Toronto, Ontario Canada; 3 Department of Clinical Biochemistry, University Health Network, 60 Murray St. Box 32, Floor 6, Rm L6-201, Toronto, ON MST 3L9 Canada

**Keywords:** Circulating tumor DNA, Cancer monitoring, Liquid biopsy, Cancer biomarkers, Cancer screening, Lung cancer

## Abstract

Advances in deep sequencing technology have led to developments in personalized medicine. Here, we describe the implications of a recent investigation that sequenced ctDNA from the plasma of non-small cell lung cancer patients to develop personalized ctDNA tests. These ‘liquid biopsies’ have shown promise in monitoring tumor growth and response to treatment, providing a timely overview of mutations present in the tumor. We discuss the advantages of this budding approach, as well as its challenges and drawbacks, while also providing areas for further investigation and an outlook for the future.

## Background

Recently, much attention has been focused on the diagnostic and therapeutic promise of the ‘liquid biopsy’. Instead of the invasive procedure of surgically removing a piece of tumor, the liquid biopsy proposes the use of tumor DNA fragments found in cancer patients’ serum, harvestable through a simple blood draw [1]. This circulating tumor DNA (ctDNA) is thought to be secreted into the bloodstream by tumor cells undergoing necrosis or apoptosis [1], and can be extracted, sequenced, and analyzed to provide information about the composition of the tumor, just like a traditional biopsy [1].

## Personalized cancer monitoring

Results from the TRACERx study using ctDNA analysis to map the evolution of early-stage lung cancer were published, to great attention, by Abbosh et al. [2] in *Nature* in May 2017. The study cohort was composed of 100 individuals diagnosed with early-stage non-small cell lung cancer (NSCLC) and undergoing surgery to remove their tumors [2]. Using the tumor samples gathered during surgery, the team began by sequencing the protein coding regions of the genome using various samples of the excised tumor, as well as sequencing comparison samples of healthy tissue [2]. The sequences from diseased and healthy tissues were subsequently compared, identifying the genetic differences, specifically the single nucleotide variants (SNV). These SNVs were then used to create a patient-specific tumor profile and to develop an individualized blood plasma test for 96 of the original 100 individuals [2]. The work performed by Abbosh et al. [2] was particularly challenging considering that the amount of ctDNA in a plasma sample is miniscule, sometimes less than 1% [3], especially in early-stage disease, and thus required a very sensitive approach. These personalized tests were then used to follow 24 patients post-surgery, in the longitudinal phase of the study, to identify whether any ctDNA remained or reappeared in their plasma after treatment, indicating relapse [2].

The investigators were able to provide a comprehensive map of cancer development for 96 individuals, testing 10–32 SNVs per person [2]. The most informative SNVs were those found early in tumor development (clonal SNVs), rather than later, due to tumor heterogeneity (subclonal SNVs). For 14 relapsing patients, the authors were also able to observe treatment resistance and recurrence of cancer from ctDNA test results at an average of 70 days (however, at more than 180 days in four patients and up to 346 days in one patient) before tumors appeared on computed tomography scans. Another useful observation was the correlation between increased frequency of SNV mutations in the ctDNA and tumor size, which allowed the investigators to extrapolate tumor size based on variant allele frequency in the sample [2].

While providing a novel way of testing for treatment success and relapse, ctDNA testing could yield many benefits to patients and clinicians, including less discomfort, more personalized treatment, and genetic profiling of tumor heterogeneity and dynamics. A glaring advantage of the liquid biopsy described in this paper is its non-invasiveness, since it can be performed using serum from a simple blood draw, a far more comfortable alternative to the needle biopsy typically used to assess lung tumors [2]. ctDNA could then be extracted from blood serum, sequenced, and analyzed, providing the clinician with insight into the genetic composition of the tumor and, in the future, informing on the most effective treatments [4].

Due to the invasiveness and discomfort of the procedure, biopsies are not often performed. However, the composition of tumors is constantly changing as new mutations occur. Thus, a patient could be receiving chemotherapy medication targeted at mutations that were relevant months or even years prior, limiting the efficacy of treatment [4].

Liquid biopsies could be performed far more often than traditional biopsies, for example, at the same time as other routine cancer bloodwork. Thus, the approach in this paper is truly pioneering because it opens the door to the continuous mapping of tumor evolution. This information will enable clinicians to prescribe chemotherapy regimes targeted specifically to the mutations currently present in their patients’ tumors.

Nevertheless, although ctDNA testing for NSCLC is very promising, it is still in its infancy. Careful consideration of detection sensitivity, cost, and clinical utility is very important as this innovation continues to be developed and refined. Presently, as the authors clearly state, this test falls short of being sensitive enough to be useable as a diagnostic marker to test for the disease [2]. Thus, it is not able to fulfill the single most important clinical need, namely detection of lung cancer in asymptomatic individuals. However, this objective was not part of the investigation.

A further challenge with ctDNA testing is cost and time. The authors estimate the combined cost of sequencing a single genome region, creating a custom tumor profile, and finally developing a liquid biopsy test, at US $1750 [2]. Moreover, the ctDNA test described in the paper is technically complex and requires specialized skills and equipment [2]; samples would likely have to be shipped to a central location, making it weeks before clinicians could receive results.

Additionally, despite requiring significant expertise and money, this test only provided an average lead time of 2 months (though one patient experienced a lead time of 344 days) [2]. Presently, no treatment exists that would change the outcome of NSCLC if given 2 months earlier, which decreases the present clinical utility of ctDNA testing for NSCLC. However, promising advances are being made in immunotherapy [5], which could improve outcomes for individuals starting this treatment even 2 months earlier as a result of a positive result on a ctDNA test. We also anticipate that technical developments may increase the current sensitivity of this test to allow even longer lead times.

A final challenge with the approach discussed is that ctDNA testing was not compared to the lung cancer circulating protein biomarkers that are also measurable in serum. For example, the measurement of CEA and CYFRA 21-1 is recommended by practice guidelines during the treatment of NSCLC, while NSE and ProGRP are recommended in small cell lung cancer treatment (https://www.researchgate.net/publication/242354126_National_Academy_of_Clinical_Biochemistry_Guidelines_for_the_Use_of_Tumor_Markers_in_Lung_Cancer). By not comparing these older biomarkers to the new ones discussed in the paper, it is difficult to determine those with the most clinical utility or whether this new, more expensive test is indeed more effective than traditional tests. Further research is needed to uncover whether traditional protein biomarkers or new ctDNA testing provides the most benefits to the clinic. It is even possible that these tests are synergistic; for example, one would be able to detect the relapsing patients missed by the other, and vice versa, to provide a more comprehensive picture of relapses.

## Conclusions

NSCLC testing using ctDNA is a promising step for precision medicine and the liquid biopsy towards a new era where continuous genetic profiling of tumors enables the most effective treatment. The approach discussed in the paper could also be further applied to other cancers, such as breast and colorectal, where ctDNA testing has already been studied [6, 7]. Careful consideration and comparison of this new method with traditional biomarkers and tests is needed to ensure ctDNA testing benefits patients and makes a smooth transition from the lab to the clinic.

## References

[1] Sirvagegna G, Marsoni S, Siena S, Bardelli A (2017). Integrating liquid biopsies into the management of cancer. Nat Rev Clin Oncol.

[2] Abbosh C (2017). Phylogenetic ctDNA analysis depicts early-stage lung cancer evolution. Nature.

[3] Diaz LA, Bardelli A (2014). Liquid biopsies: genotyping circulating tumor DNA. J Clin Oncol.

[4] Bardelli A (2017). Personalized test tracks cancer relapse. Nature.

[5] Farkona S, Diamandis EP, Blasutiq IM (2016). Cancer immunotherapy: the beginning of the end of cancer?. BMC Med.

[6] Garcia-Murillas I (2015). Mutation tracking in circulating tumor DNA predicts relapse in early breast cancer. Sci Transl Med.

[7] Tie J (2015). Circulating tumor DNA as an early marker of therapeutic response in patients with metastatic colorectal cancer. Ann Oncol.

